# Influence of Laser Process Parameters on the Forming Quality and Discharge Performance of 3D-Printed Porous Anodes for Al–Air Batteries

**DOI:** 10.3390/ma17122837

**Published:** 2024-06-11

**Authors:** Keqing Wang, Zheming Hu, Chutong Yin, Shuangchi Qin, Peng Li, Jiahui Guan, Kui Zhu, Yin Li, Sida Tang, Jitai Han

**Affiliations:** 1 School of Automation, Wuxi University, Wuxi 214105, China; keqing_w2023@163.com (K.W.); zmhu@stu.cwxu.edu.cn (Z.H.); tcyin@stu.cwxu.edu.cn (C.Y.); scqin@stu.cwxu.edu.cn (S.Q.); tomtsd@163.com (S.T.);; 2 School of Automation, Nanjing University of Information Science and Technology, Nanjing 210044, China; 3 Hongyuan Green Energy Co., Ltd., Wuxi 214128, China

**Keywords:** Al–air batteries, 3D printing, SLM, printing parameters, discharge characterization

## Abstract

Aluminum–air (Al–air) batteries are considered one of the most promising next-generation energy storage devices. In this paper, we carry out an orthogonal experimental study on the SLM printing process parameters in 3D-printed Al–air battery anodes. The surface roughness, densification, and discharge performance of the electrodes under different process parameters are observed to reveal the effects of different process parameters on the forming quality and discharge performance of aluminum–air battery anodes. The results show that the laser power is the most important factor affecting the surface roughness of the porous aluminum anode, and the scanning spacing is the most important factor affecting the densification. The best printing parameters for the porous aluminum anode can be obtained when the laser power is 325 W, the scanning speed is 1000 mm/s, the scanning spacing is 0.12 mm, and the thickness of the powder spread is 0.03 mm. At this time, the surface roughness of the porous aluminum anode obtained by this process parameter is 15.01 μm, the densification is 94.97%, and the discharge is stable with a high value. In addition, we also carry out data validation to ensure that the data we obtain are optimal and valid.

## 1. Introduction

With the growing demand for safe and sustainable energy, over the past few decades, a variety of new clean energy sources and energy storage systems have emerged and been fully developed [1,2,3,4,5]. Among them, aluminum–air batteries (a new type of energy device) usually use metal aluminum anodes as the raw material and oxygen from the air as the combustion agent. A catalyst on the cathode allows for the conversion of the metal’s own chemical energy into electrical energy. It has the advantages of high theoretical potential, abundant mineral resources, safe use, and green non-pollution. Therefore, it has received wide attention in the fields of energy storage power supply, new energy vehicles, aerospace, and military supplies [1,2,6,7,8].

However, although the aluminum–air battery system is considered the most promising battery technology for low-cost high-performance rechargeable batteries, it still needs to be perfected to develop into a battery technology with fast preparation and molding and stable discharge [9,10,11]. This is mainly attributed to the fact that the current preparation of aluminum anodes mainly adopts traditional metal forming methods such as rolling, cold working, and deformation. The preparation time often takes 2 to 3 h, which is long. The problems of low anode utilization and improved discharge capacity have not been solved [12,13,14]. For this reason, many researchers have applied 3D printing technology to the application of aluminum–air batteries in recent years [15,16,17,18,19,20]. Yu prepared porous aluminum anodes using SLS, which still had poor discharge efficiency. The reason for this is that the added organic solvents, such as rosinol and ethyl cellulose, did not completely evaporate, thus reducing the conductivity of the aluminum anode [21,22]. Although Jiang et al. optimized the results, there is still room for improvement [23]. Han prepared flat plate aluminum anodes using the SLM process. However, due to the improper selection of process parameters, the surface of the aluminum anode had many macroscopic defects and a high self-corrosion rate. Therefore, the same problem of poor discharge performance existed [24]. It can be seen that, although 3D printing technology has shown significant advantages in rapid prototyping and other aspects (promoting the application research of aluminum–air batteries), the size of the laser process parameters directly determines the melting state of the metal powder, which in turn affects the temperature of the molten pool. This affects the surface quality of the porous aluminum anode and ultimately the discharge performance of the aluminum–air battery. For this reason, it is crucial to study the reasonable printing process parameters to improve the forming quality and discharge performance of the electrodes.

In this paper, we firstly used orthogonal experiments to refine the one-factor action intervals by preferentially selecting the main influencing parameters (laser power, scanning speed, scanning spacing, and powder laying thickness) in the printing process parameters. Second, three-dimensional morphology and metallographic verification of the electrode surface roughness and densities in the forming quality were performed. This information was then used to look into how well aluminum–air batteries discharged under different conditions. In addition to this, we carried out data validation and proper mechanism deduction based on the optimal printing process parameters to ensure that the data we obtained were optimal and valid. The results obtained in this study provide an effective reference for other researchers to further improve the overall performance of aluminum–air batteries.

## 2. Experiment

### 2.1. Material Preparation

**Figure 1:**
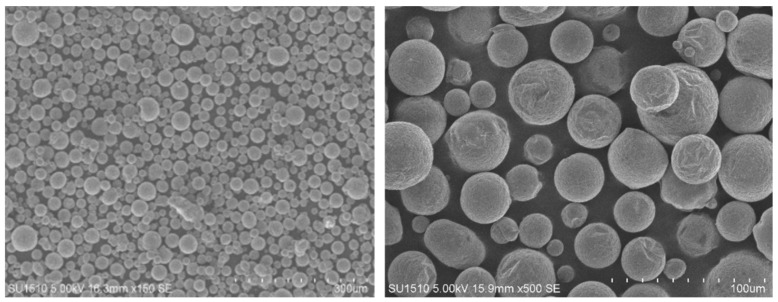
Microscopic morphology of 6061 aluminum alloy powder after milling.

The original 6061 aluminum alloy powder, production lot number G2019ZFGA010, was from AVIC Gighty Powder Metallurgy Technology (Beijing, China) Co. Given the importance of powder quality in determining the quality of 3D-printed parts, a combination of low-speed ball milling and vacuum drying was used to process the original powder. The particle morphology of the material powder after ball milling is shown in Figure 1.

### 2.2. Instruments

XDM 250 type under the powder supply type laser additive manufacturing equipment for SLM forming of composite aluminum alloy material was selected from Xidimo 3D Printing Technology Company (Suzhou, China). The external dimensions of the equipment were 1670 mm × 1140 mm × 2160 mm. The maximum forming size was 250 mm × 250 mm × 410 mm, equipped with F-Theta mirrors, a high-speed and high-precision scanning head, and a single-mode fiber laser. The machine was equipped with an F-Theta mirror, a high-speed and high-precision scanning head, a single-mode fiber laser, oxygen content below 100 ppm to prevent high-temperature oxidation reaction of the powder, and argon as the inert protective gas.

At the end of the print molding, the molded prototypes were cut by EDM and the surface morphology of the printed anodes was observed using a three-dimensional profiler (MFP-D, RTEC, Bruker, Billerica, MA, USA) and a scanning electron microscope (SU1510, Hitachi, Tokyo, Japan). We used the LANHE battery test system (CT3001A) from Wuhan Lanhe Electronics Co., Ltd. (Wuhan, China), and the CHI750E electrochemical workstation from Shanghai Chenhua Instrument Co., Ltd. (Shanghai, China), to check how well the battery system discharged and how well it worked electrically.

### 2.3. Experimental Program

The quality of SLM forming serves as an important factor affecting the discharge performance of aluminum–air batteries. About 13 of these parameters can have an impact on the final performance of the formed part [25,26,27,28]. Laser power, scanning speed, scanning pitch, and powder thickness are relatively important and widely studied parameters. Therefore, in this paper, a four-factor three-level orthogonal experiment was designed to investigate the effects of four process parameters, namely laser power, scanning speed, scanning spacing, and powder thickness, on the forming quality of porous aluminum anodes. The orthogonal experimental design is shown in Table 1.

**Figure 2:**
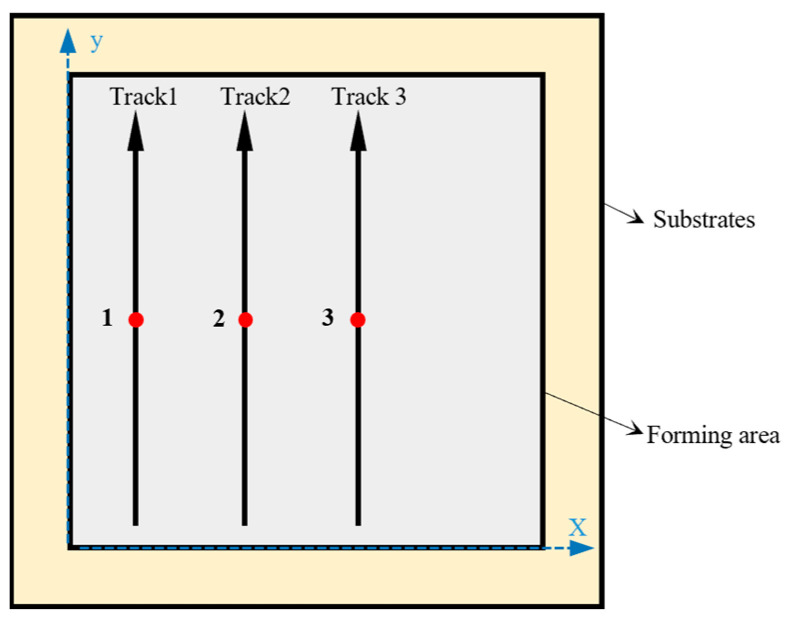
Schematic diagram of SLM scanning strategy. Where 1, 2, and 3 in the figure are the midpoints of three different scanning trajectories, respectively.

At the same time, the biggest difference between the same scanning mold strategies is the severity of remelting. This makes the cooling rate and localized heat treatment different. For this reason, the experiments in this paper were carried out uniformly using linear scanning, as shown in Figure 2.

**Figure 3:**
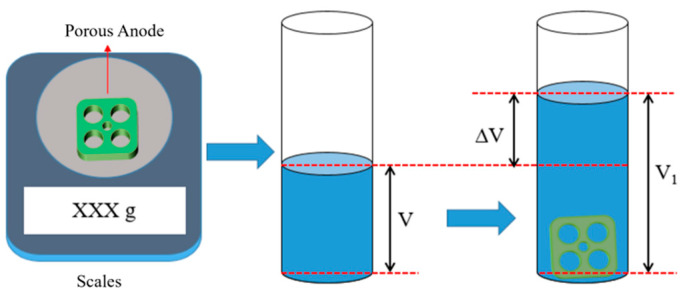
Schematic diagram of density test by drainage method.

The surface quality of the specimen (surface roughness, densification, etc.) is particularly important for its performance. Poor surface quality not only reduces the corrosion resistance of porous aluminum anodes but also may cause a decrease in the discharge performance of aluminum–air batteries. The four 3D printing process parameters can change how well porous aluminum anodes work. This is because of the laser energy density. The relationship between laser energy density and process parameters is as follows:

(1) $$E=\frac{P}{V\cdot T\cdot D}$$

where E is the energy density, J/mm^3^; P is the laser power, W; V is the scanning speed, mm/s; T is the powder thickness, mm; D indicates the scanning interval, mm. In addition, the densification of porous aluminum anodes was tested using the drainage method, the principle of which is shown in Figure 3. The formula for calculating the densities by the drainage method is as follows:

(2) ψ=1−MV1−V0ρ·100%

where ψ is the density, %; M is the mass of porous aluminum anode, g; $V_{0}$ and $V_{1}$ are the volume of water in the measuring cylinder before and after the sample is placed, mL; ρ is the density of water, g/cm^3^.

## 3. Results and Discussion

### 3.1. Influence of Process Parameters on Forming Quality

Different combinations of 3D printing process parameters firstly affect the forming quality of porous aluminum anodes, such as surface roughness and densification. This in turn affects the discharge performance of aluminum–air batteries. For this reason, it is necessary to carry out a study on the influence of 3D printing process parameters on the forming quality of aluminum–air batteries. In this section, the test results of surface roughness and densification of porous aluminum anodes are listed, as shown in Table 2.

#### 3.1.1. Surface Roughness

Table 3 shows the calculation results of the extreme difference of surface roughness. From this table, it can be seen that the magnitude of the extreme deviation of the four factors is RA > RB > RD > RC., i.e., among the four parameters of 3D printing, the laser power has the greatest influence on the surface roughness of the porous aluminum anode, followed by scanning speed and scanning spacing. The last is the thickness of the powder laying. Combining the three K-values of the four factors gives the optimum process parameter combination of A3B2D2C2. This suggests that the optimum process parameter combination is P = 325 W, V = 1000 mm/s, D = 0.12 mm, T = 0.03 mm.

**Figure 4:**
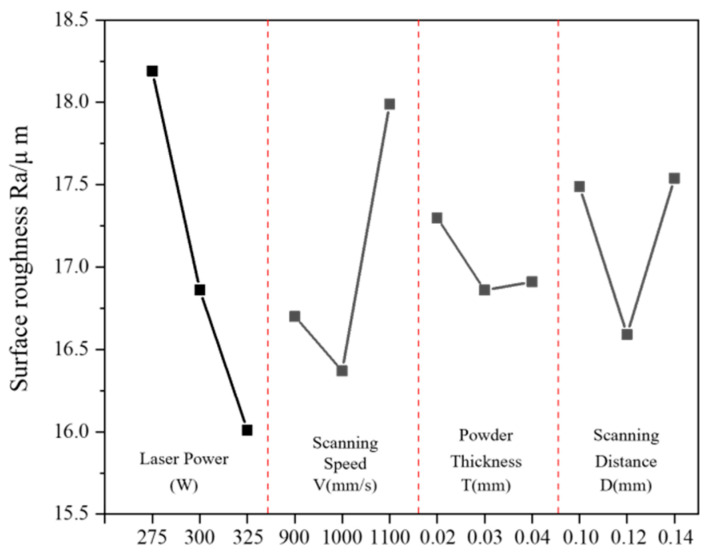
Influence of different factors on the surface roughness.

Figure 4 shows the influence curve of different factors on the surface roughness in the porous aluminum anode. The relationship between the four process parameters and the surface roughness in the porous aluminum anode can be further analyzed based on this figure. When the range of laser power is 275 W~325 W, the surface roughness decreases gradually with the increase in laser power. At the same time, the surface roughness basically decreases almost linearly with the increase in laser power. The surface roughness decreases from 18.19 μm to 16.86 μm and 16.01 μm, with 7.31% and 11.98%, respectively. The effects of scanning speed on the electrode surface roughness all show a trend of decreasing and then increasing. Meanwhile, the variation of surface roughness in the interval of 1000 m/s~1100 m/s is significantly higher than that in the front part. The trends of the effects of powder laying thickness and scanning spacing on surface roughness are similar to those of scanning speed, while showing a smaller variation amplitude.

#### 3.1.2. Homogeneity

Table 4 shows the calculation results of the extreme difference of densities. From this table, it can be seen that the magnitude of the extreme difference of the four factors is RD > RA > RC > RB. That is to say, among the four process parameters of 3D printing, the order of influence on the densities is scanning pitch > laser power > powder thickness > scanning speed. Combining the three K-values of the four factors, the optimal process parameter combination can be obtained as D2A3C3B3 because the optimal process parameter combination indicated here is D = 0.12 mm, P = 325 W, V = 1100 mm/s, T = 0.04 mm.

**Figure 5:**
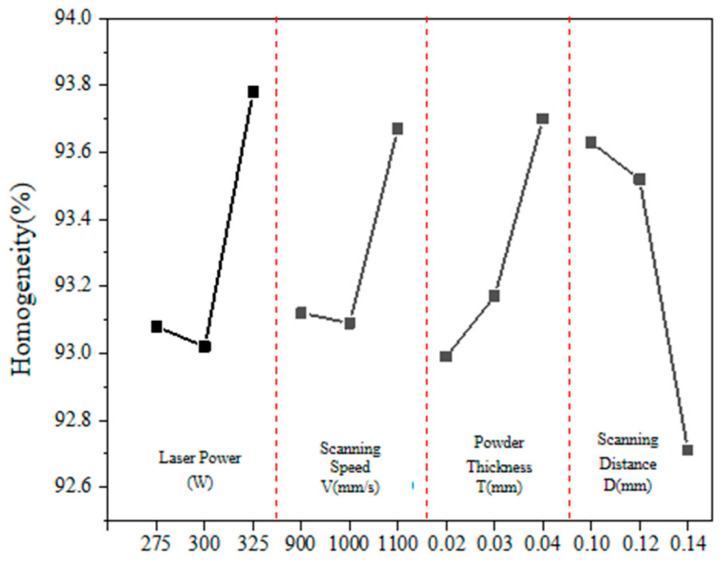
Influence of different factors on the density.

Figure 5 shows the influence curve of different factors on the porous aluminum anode density. According to this figure, the relationship between the four process parameters and the porous aluminum anode densities can be further analyzed. The density of the porous anode increases with increasing laser power. This indicates that, with the increase in laser power, the densities of the specimens show a trend of slightly decreasing and then increasing. The effects of scanning speed and powder thickness on the densities of the specimens are similar to those of the laser power. That is, with the increase in scanning speed, the density of the specimen firstly decreases and then increases. The scanning spacing is different from the former, showing a linear decreasing trend. This indicates that the effect of scanning distance on the densities is opposite to that of the thickness of the laid powder. At the same time, it is not difficult to see that, when the thickness of the laid powder is in the range of 0.10 mm~0.12 mm, the value of the densification is relatively stable. When the sweep spacing is 0.14 mm, the densification of the porous aluminum anode decreases sharply to only 92.71%. This indicates that the variation of the sweep thickness has a more direct effect on the molding effect of the densities.

Based on the results of the above orthogonal experiments carried out, it is not difficult to obtain the following summary inferences. For the laser power, the energy absorbed by the aluminum alloy powder increases with the increase in laser power in the process of 3D printing, forming the porous aluminum anode. When the laser power is small, the energy acting on the aluminum alloy powder is not enough to completely melt the powder. When the dip angle of the molten pool becomes large, it tends to cause spheroidization. This tends to cause unevenness in the formed surface. At the same time, as this phenomenon accumulates layer by layer, it eventually leads to an increase in surface roughness and a decrease in the densification of the aluminum anode. As the laser power increases, the energy applied to the aluminum alloy powder increases and the powder melts more fully. The metallurgical bonding effect between adjacent channels and adjacent layers is significantly improved. As a result, the surface roughness of the aluminum anode gradually decreases and the density increases. And, when the laser power is too high, the aluminum alloy powder absorbs too much energy. The powder is prone to overcooking and splashing. This affects the metallurgical bonding between adjacent fusion channels and adjacent layers. Thus, the surface roughness and densification of the porous aluminum anode are affected. When the scanning speed is small, the laser stays on the aluminum alloy powder for a long time. The amount of energy that the aluminum alloy powder absorbs per unit of time is higher. The dip angle of the molten pool is smaller, the spheroidization is weak, and the powder melts more fully. The forming surface is relatively flat. As a result, the surface roughness and densification of the porous aluminum anode is relatively good. The increase in scanning speed shortens the time that the laser acts on the aluminum alloy powder. The reduction of energy absorbed by the powder tends to cause adjacent fusion channels and the binding ability between the upper and lower layers. The surface roughness and densification of the specimen are affected. When the thickness of the powder is small, the energy acting on the aluminum alloy powder is large. Therefore, the powder melting is more adequate, and the dip angle of the melt pool is small. As a result, the formed surface is flatter. When the thickness of the powder layer increases, the energy input on the metal powder decreases. The laser energy melts the powder layer to a limited depth. The lower layer of powder is not completely melted and balling tends to increase and thus the thermal conductivity deteriorates. As the printing process continues, the accumulation of the thickness of the layered powder further reduces the thermal conductivity. This results in changes in the surface roughness and densification of the specimen. When the scanning distance is too small, the overlap between adjacent channels is too large and the laser energy applied to the aluminum alloy powder is high. The remelting expansion region of the channels is significantly higher than the height of a single channel. It is easy to cause overburning, leading to spheroidization. The surface roughness of the aluminum anode increases and the densification decreases. When the scanning spacing is too large, the overlap between adjacent channels is reduced or non-existent. It is difficult to form a smooth plane between the channels. The same leads to an increase in surface roughness and a decrease in densification. Therefore, suitable 3D printing parameters are favorable for the overlap between the channels and the energy input. The aluminum alloy powder is more fully melted and the surface roughness and densification of the porous aluminum anode is relatively good.

According to the results of orthogonal experiments, laser power is the most important factor affecting the surface roughness of the porous aluminum anode. The scanning pitch is the most important factor affecting the densification. The optimal set of parameters for the 3D printing porous aluminum anode is obtained as P = 325 W, V = 1000 mm/s, D = 0.12 mm, T = 0.03 mm, and the surface roughness of the aluminum anode is 15.01 μm, and, by using this optimal set of process parameters, the densification is 94.97%. It can be found that the surface roughness and density of the porous aluminum anode formed under this group of optimal process parameter combinations are better than those of the above nine process parameter combinations.

**Figure 6:**
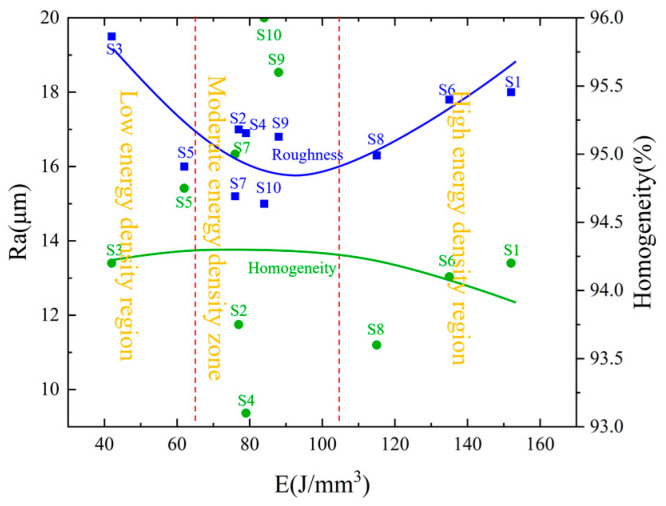
Influence of laser energy density on surface roughness and density.

The effect of orthogonal experimental parameter combinations on the quality of porous aluminum anode forming can be attributed to the laser energy density. To facilitate the understanding of the relationship between laser energy density and surface roughness and densification, Figure 6 shows the variation of surface roughness and densification as a function of laser energy density.

Figure 6 shows a plot of surface roughness and densification versus laser energy density. The overall trend of this figure shows that with, the increase in laser energy density, surface roughness shows a tendency to decrease and then increase, and densification also shows a tendency to decrease and then increase.

### 3.2. Influence of Molding Quality on Discharge Properties

The above analyses indicate the variation of surface roughness and densification in the forming quality of porous aluminum anodes in terms of individual process parameters in orthogonal experiments. At the same time, we obtain the optimal process parameters for the anodes of 6061 alloy for aluminum–air batteries by conducting the above experimental studies. For this reason, in the next section, we will further investigate the effect of the forming quality of porous aluminum anodes on the discharge voltage, anode utilization, and specific capacity of aluminum–air batteries for different 3D printing process parameters. To facilitate the analysis, S is added in front of the first column of numbers in Table 1 to represent specimens 1–9, respectively. The porous aluminum anode formed by the combination of the optimum set of process parameters is named S10.

#### 3.2.1. Discharge Performance

**Figure 7:**
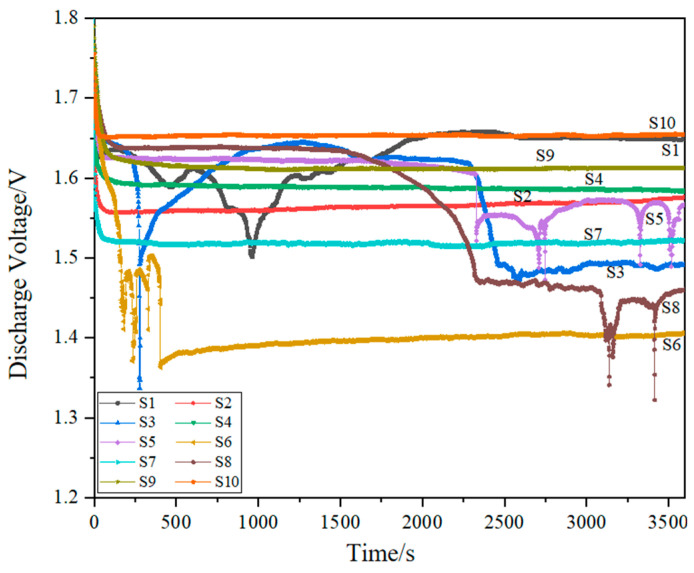
Discharge voltage curves of porous Al anodes at different process parameters.

Figure 7 shows the discharge voltage curves of the porous aluminum anode under different process parameters. From this figure, it can be seen that the discharge voltage curves of aluminum–air batteries under different combinations of 3D printing parameters can be divided into two cases—stable discharge and non-stable discharge. The discharge voltage of the aluminum–air battery consisting of porous aluminum anode specimens S1, S3, S5, S6, and S8 fluctuates greatly during the discharge process, while the discharge voltage of the aluminum–air cell consisting of specimens S2, S4, S7, S9, and S10 is relatively stable during the discharge process. Combined with Figure 6, it is found that the variation of laser energy density causes the difference in the discharge voltage curve of the aluminum–air battery. Based on the variation of the discharge voltage curves, the laser energy density is divided into three intervals as follows: the low energy density interval (<70 J/mm^3^), the moderate energy density interval (70–100 J/mm^3^), and the high energy density interval (>100 J/mm^3^). It is found that the discharge voltages of aluminum–air batteries consisting of porous aluminum anodes in the low energy density interval (S3, S5) and high energy density interval (S1, S6 and S8) are all non-stationary discharges, whereas the aluminum–air batteries consisting of porous aluminum anodes within the moderate energy density interval (S2, S4, S7, S9, and S10) are all stable discharges.

**Figure 8:**
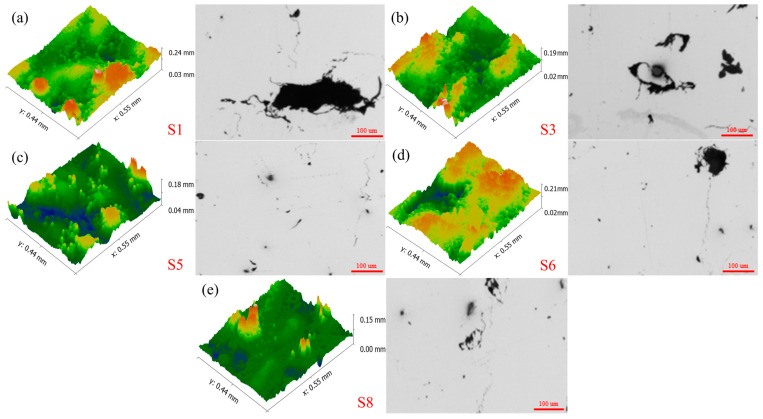
(**a**) High-energy-density interval sample S1 3D morphology and metallograph; (**b**) Low-energy-density interval sample S3 3D morphology and metallograph; (**c**) Low-energy-density interval sample S5 3D morphology and metallograph; (**d**) High-energy-density interval sample S6 3D morphology and metallograph; (**e**) High-energy-density interval sample S8 3D morphology and metallograph.

Figure 8 shows the 3D morphology and metallography of the porous aluminum anode in the low and high energy density intervals. When the laser energy density is 44.64 J/mm^3^, the input energy is small at this time. The energy acting on the aluminum alloy powder is not enough to completely melt the powder, which easily causes defects such as unmelted powder and holes. This results in a larger surface roughness and lower densification of the porous aluminum anode. During the discharge process, the presence of unmelted metal powder and holes causes an uneven reaction on the surface of the porous aluminum anode. The non-uniformity of the reaction exacerbates the local potential difference on the anode surface, thus leading to instability in the discharge process. With the increase in laser energy density, the discharge type of aluminum–air battery is a stable discharge. At this time, the surface roughness of the porous aluminum anode is relatively low and the densification is high. When the laser energy density increases further, the discharge type of the aluminum–air cell changes to a non-stationary discharge. In the high energy density interval, the laser acts on the aluminum alloy powder with too much energy. This leads to overcooking and spattering of the aluminum alloy powder, resulting in defects such as porosity on the surface. Large surface roughness and low densities also contribute to the instability of the aluminum–air battery discharge process.

**Figure 9:**
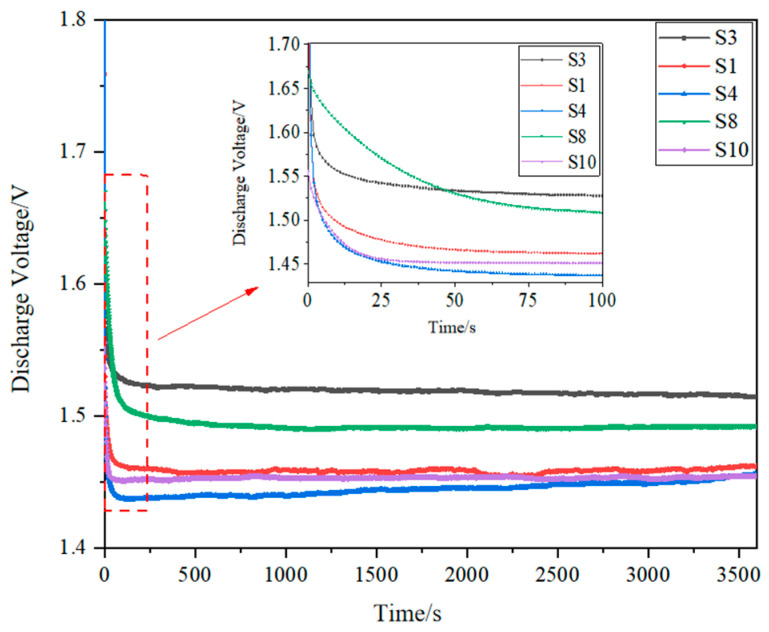
Discharge voltage curves of porous Al anodes at moderate energy density range.

**Figure 10:**
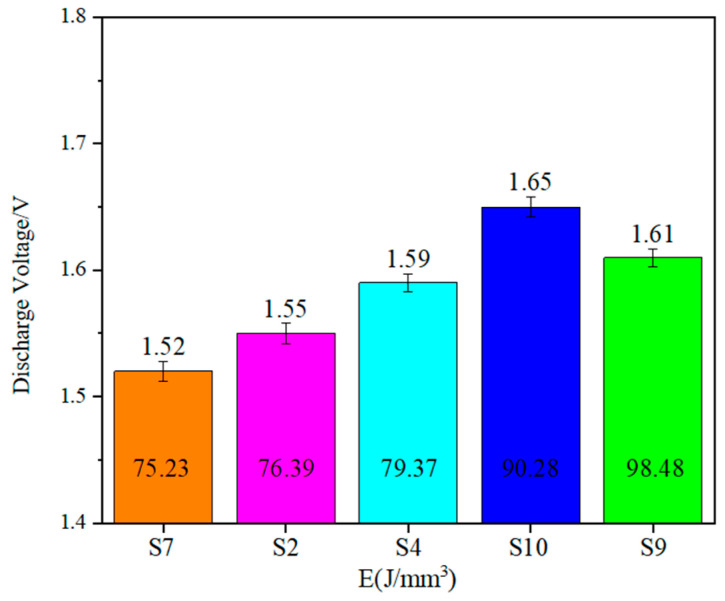
Effect of laser energy density on discharge voltage.

The discharge voltage curves in Figure 9 show that the discharge is not always stable when porous aluminum anodes are formed in the low energy density interval and the high energy density interval to make an aluminum–air battery. Therefore, the specimen formed within this interval is not suitable as an anode for aluminum–air batteries, whereas the discharge stability of aluminum–air batteries composed of porous aluminum anodes formed within the moderate energy density interval is higher. Therefore, the specimens formed in this interval can be used as anodes for aluminum–air batteries. The variation of the discharge voltage profile is a result of the combined effect of the surface roughness and densification of the porous aluminum anode. In order to study the effect of laser energy density on the discharge voltage more clearly, the discharge voltage curve of the aluminum–air battery in the interval of moderate energy density and the variation of the discharge voltage with the laser energy density are made, as shown in Figure 9 and Figure 10.

Figure 10 shows the effect of laser energy density on the discharge voltage. From the figure, it can be seen that the discharge voltage of the aluminum–air battery is 1.52 V when the laser energy density is 75.23 J/mm^3^. When the laser energy density is 76.39 J/mm^3^ and 79.37 J/mm^3^, the discharge voltage increases by 1.97% and 4.61%, respectively. When the laser energy density is further increased to 90.28 J/mm^3^, the discharge voltage is maximum at 1.65 V. And when the laser energy density is increased to 98.48 J/mm^3^, the discharge voltage decreases by 2.42%. The overall trend shows that, with the increase in laser energy density, the discharge voltage shows a tendency of increasing and then decreasing. This is the result of the combined effect of surface roughness and densification of the porous aluminum anode.

#### 3.2.2. Anode Utilization

**Figure 11:**
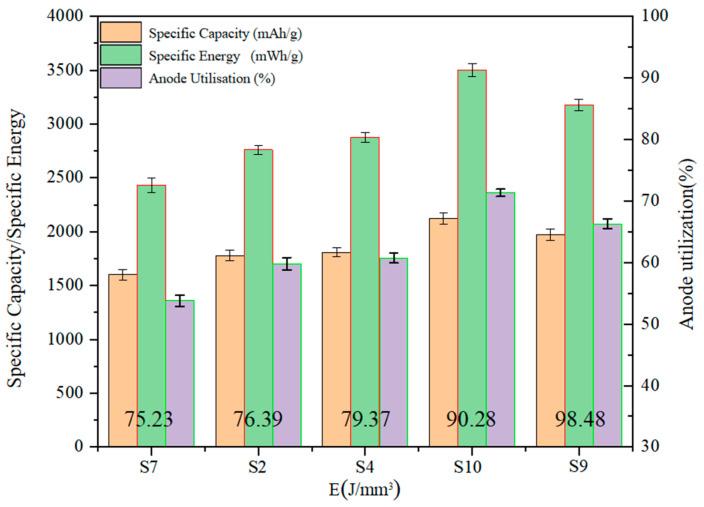
Discharge performance of Al–air battery at different laser energy densities.

Figure 11 shows the variation of anode utilization for aluminum–air batteries at different laser energy densities. It can be seen that the anode utilization rate shows an increasing and then decreasing trend with the increase in laser energy density. When the laser energy density is 75.23 J/mm^3^, the anode utilization is 53.79%. When the laser energy density increases to 76.39 J/mm^3^ and 79.37 J/mm^3^, the anode utilization rate increases by 11.23% and 12.96% at this time, respectively. When the laser energy density increases to 90.28 J/mm^3^, the anode utilization at this point is maximum at 71.32%. Compared with the S7 aluminum anode at a laser energy density of 75.23 J/mm^3^, the anode utilization increases by 32.59%. However, when the laser energy density is 98.48 J/mm^3^, the anode utilization decreases by 7.08%. The magnitude of the values of the specific capacity and specific energy of the aluminum–air battery is affected by the calculation formula, and the trend of change presents a consistent feature with the anode utilization rate.

**Figure 12:**
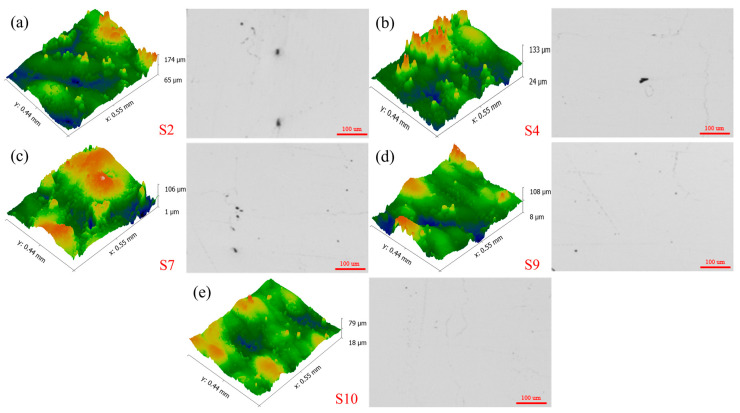
Surface morphology of S2, S4, S7, S9, S10 (**a**–**e**) porous aluminium anodes in the medium energy density range.

It is easy to see from Figure 10 and Figure 11 that, in the moderate energy density interval, with the increase in laser energy density, the discharge voltage, anode utilization, specific capacity, and specific energy of the aluminum–air battery all show a tendency to increase first and then decrease. This is a result of the combined effect of surface roughness and densification of the porous aluminum anode. As shown in Figure 12, when the laser energy density is low, the porous aluminum anode has poor forming quality and high surface roughness. Therefore, the potential difference on the anode surface is large. At the same time, the lower densities accelerate the non-uniform corrosion of the anode to some extent. When the laser energy density is too high, the higher surface roughness and lower densities also cause the degradation of the discharge performance. Lower surface roughness and higher densities can reduce the potential difference and corrosion non-uniformity on the anode surface and improve the discharge performance of the aluminum–air battery.

#### 3.2.3. Electrical Discharge Pattern

**Figure 13:**
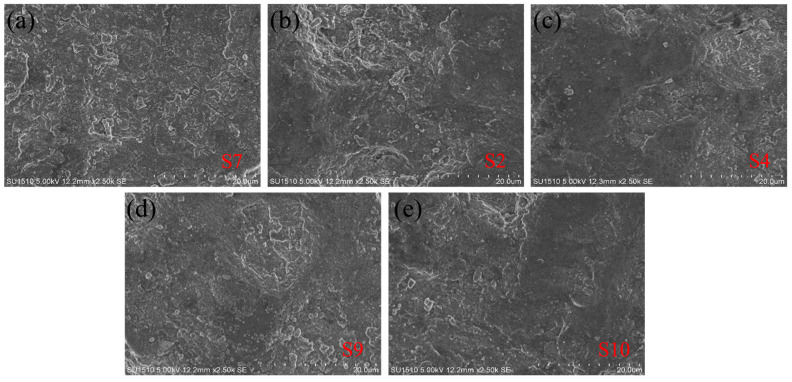
(**a**) Scanning electron micrograph of S7 after discharge; (**b**) Scanning electron micrograph of S2 after discharge; (**c**) Scanning electron micrograph of S4 after discharge; (**d**) Scanning electron micrograph of S9 after discharge; (**e**) Scanning electron micrograph of S10 after discharge.

To further analyze the effect of laser energy density on the discharge performance of porous aluminum anode, the surface morphology of the porous aluminum anode in the 20 µm state after discharge is examined using a scanning electron microscope, as shown in Figure 13. From this figure, it can be seen that the discharge surface of S7 and S2 porous aluminum anodes is relatively rough at the end of the discharge in the moderate energy interval. The surface is accompanied by obvious local corrosion, and corrosion pits can be observed, while the discharge surfaces of S4, S9, and S10 porous aluminum anodes are relatively flat and the local corrosion phenomenon is obviously reduced. In other words, with the reduction in anode surface roughness and the improvement in densification, the degree of corrosion is gradually reduced and the corrosion resistance is improved. For porous aluminum anodes, both the discharge and corrosion during the whole discharge process need to consume anode material. When the corrosion rate is small, this means that the anode mainly undergoes a discharge reaction. Therefore, porous aluminum anodes with low surface roughness and high densities show higher discharge performance.

#### 3.2.4. Validation of Validity

**Figure 14:**
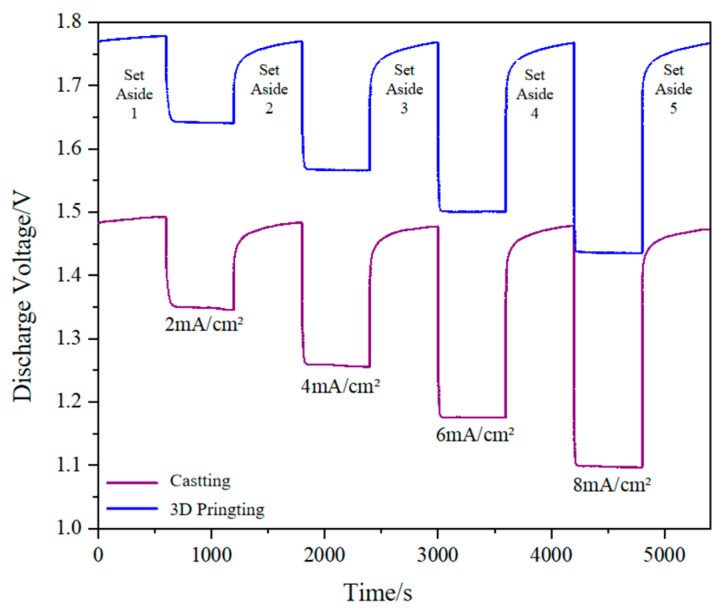
Discharge curve of 3D printing and casting Al anode at different current densities.

Figure 14 shows the discharge curves of 3D-printed and cast aluminum anodes under different discharge current densities. It can be seen that, for aluminum anodes prepared by different processes, the discharge voltage of the aluminum–air battery is basically stable under different discharge current densities, and the whole discharge process is a cycle of static stabilization/declining discharge/stable discharge/restoring static. Table 5 shows the constant current discharge results under different discharge current densities. It can be seen that the discharge voltage from 3D-printed aluminum anodes is higher than that of cast aluminum anodes at different resting stages and different discharge current densities. The discharge voltage of the 3D-printed aluminum anode is 20.27% higher than that of the cast aluminum anode at different static stages, whereas, at discharge current densities of 2, 4, 6, and 8 mA/cm^2^, the discharge voltages of 3D-printed aluminum anodes are increased by 23.13%, 25.39%, 27.12%, and 29.46%, respectively. This shows that the 3D printing process can significantly improve the discharge performance of aluminum–air batteries compared with the traditional process. The process printing parameters serve as the key to the electrode forming quality, therefore they have a direct impact on the discharge performance of aluminum–air batteries. This shows that our experiment is carried out effectively and has practical research value.

## 4. Conclusions

In this paper, the effect of process parameters on the forming quality of porous aluminum anodes was investigated by using orthogonal experiments. We looked at how surface roughness and densification affect the ability of aluminum–air batteries to discharge, along with laser energy density. Meanwhile, the surface roughness model and discharge performance model were established on this basis. And the discharge performance of aluminum anodes prepared by different processes were compared. Through the comparison, it was not difficult to understand that, due to the interaction between the laser beam and the material, the laser power directly affects the melting of the material and the surface, forming smoothness. Therefore, laser power and scanning spacing are the most important factors affecting the surface roughness and densification of the porous anode, respectively. The porous aluminum anode-forming quality can reach the optimal value when the laser process parameters are P = 325 W, V = 1000 mm/s, D = 0.12 mm, T = 0.03 mm, respectively. In addition, the derivation of model theoretical algorithms successfully confirmed that the process parameters can indeed influence the anode surface-forming quality and discharge performance of aluminum–air batteries.

## Figures and Tables

**Table 1:** Experimental program of scanning parameters.

Sequences	Laser Power p/W	Scanning Speed v/mm·s^−1^	Scanning Distance s/mm
1	260, 280, 300, 320, 340	1000	0.13
2	300	800, 900, 1000, 1100, 1200	0.13
3	300	1000	0.09, 0.11, 0.13, 0.15, 0.17

**Table 2:** Surface roughness and density value of Al anode.

Ordinal Number	Laser Power P (W)	Scanning Speed V (mm/s)	Powder Thickness T (mm)	Scanning Distance D (mm)	Surface Roughness Ra (μm)	Homogeneity (%)
1	275	900	0.02	0.10	18.05	93.19
2	275	1000	0.03	0.12	16.95	92.75
3	275	1100	0.04	0.14	19.58	93.29
4	300	900	0.03	0.14	16.89	92.15
5	300	1000	0.04	0.12	16.01	91.81
6	300	1100	0.02	0.10	17.68	93.09
7	325	900	0.04	0.12	15.15	94.01
8	325	1000	0.02	0.14	16.16	92.70
9	325	1100	0.03	0.10	16.73	94.62

**Table 3:** Range calculation results of surface roughness.

Influencing Factors	Laser Power (A)	Scanning Speed (B)	Powder Thickness (C)	Scanning Distance (D)
K1	18.19	16.70	17.30	17.49
K2	16.86	16.37	16.86	16.59
K3	16.01	17.99	16.91	17.54
R	2.18	1.62	0.44	0.95

**Table 4:** Calculation results of density range.

Influencing Factors	Laser Power (A)	Scanning Speed (B)	Powder Thickness (C)	Scanning Distance (D)
K1	93.08	93.12	92.99	93.63
K2	93.02	93.09	93.17	93.52
K3	93.78	93.67	93.70	92.71
R	0.76	0.58	0.71	0.92

**Table 5:** Discharge results of 3D printing and casting Al anode at different current densities.

Battery Anode	Discharge Current Density (mA/cm^2^)								
	Set Aside 1	2	Set Aside 2	4	Set Aside 3	6	Set Aside 4	8	Set Aside 5
3D Printing	1.78	1.65	1.78	1.58	1.78	1.50	1.78	1.45	1.78
Casting	1.48	1.34	1.47	1.26	1.47	1.18	1.47	1.12	1.46

## Data Availability

Data is contained within the article.
